# Supplementary material for: The Poisson distribution model fits UMI-based single-cell RNA-sequencing data
Source: BMC Bioinformatics. 2023 Jun 17;24:256. doi: 10.1186/s12859-023-05349-2 (PMC10276395; doi:10.1186/s12859-023-05349-2)
Supplement: Supplementary file 1 — Additional file 1. The summary of goodness-of-fit measures, including the Kolmogorov-Smirnovtest, the over-dispersion test, and the zero-inflation test. [file 12859_2023_5349_MOESM1_ESM.pdf]

Table S1: Summary of p-values from Kolmogorov-Smirnov (KS) test

Plate	L	$\lambda = 0.1$	$\lambda = 0.5$	$\lambda = 1$	$\lambda = 2$	$\lambda = 5$	$\lambda = 20$
Plate01	10	p = 1	p = 1	p = 1	p = 1	p = 1	p = 0.95
Plate03	10	p = 1	p = 1	p = 1	p = 1	p = 1	p = 0.8
Plate05A	10	p = 1	p = 1	p = 1	p = 1	p = 0.95	p = 0.94
Plate05B	10	p = 1	p = 1	p = 1	p = 1	p = 1	p = 0.25
Plate06A	10	p = 1	p = 1	p = 1	p = 1	p = 0.99	p = 0.3
Plate08	10	p = 1	p = 1	p = 1	p = 0.69	p = 1	p = 0.31
Plate08	15	p = 1	p = 1	p = 1	p = 1	p = 0.98	p = 0.47
Plate05A, Plate06A	10	p = 1	p = 0.96	p = 1	p = 1	p = 1	p = 0.07
Plate05A, Plate06A	20	p = 1	p = 1	p = 1	p = 1	p = 0.94	p = 0.9

*For large Poisson parameter, relatively fewer matrix entries are aggregated to reduce variation in the underlying parameter.

Table S2: Summary of p-values from Over-Dispersion test

Plate	L	$\lambda = 0.1$	$\lambda = 0.5$	$\lambda = 1$	$\lambda = 2$	$\lambda = 5$	$\lambda = 20$
Plate01	10	p = 0.963	p = 1	p = 0.76	p = 0.735	p = 0.194	p = 0.143
Plate03	10	p = 0.199	p = 0.988	p = 0.907	p = 0.775	p = 0.381	p = 0.215
Plate05A	10	p = 0.694	p = 0.33	p = 0.77	p = 0.395	p = 0.999	p = 0.22
Plate05B	10	p = 0.467	p = 0.992	p = 0.678	p = 0.186	p = 0.053	p = 0.062
Plate06A	10	p = 0.467	p = 0.741	p = 0.989	p = 0.801	p = 0.152	p = 0.152
Plate08	10	p = 0.981	p = 0.345	p = 0.747	p = 0.112	p = 0.147	p = 0.038
Plate08	15	p = 0.995	p = 0.918	p = 1	p = 0.391	p = 0.694	p = 0.164
Plate05A, Plate06A	10	p = 0.993	p = 0.013	p = 0.37	p = 0.514	p = 0.73	p = 0.026
Plate05A, Plate06A	20	p = 0.981	p = 0.988	p = 0.859	p = 0.645	p = 0.999	p = 0.177

*For large Poisson parameter, relatively fewer matrix entries are aggregated to reduce variation in the underlying parameter.

Table S3: Summary of p-values from Zero-Inflation test

Plate	L	$\lambda = 0.1$	$\lambda = 0.5$	$\lambda = 1$	$\lambda = 2$	$\lambda = 5$	$\lambda = 20$
Plate01	10	p = 0.055	p = 0.4	p = 0.484	p = 0.368	p = 0.126	p = 0.5
Plate03	10	p = 0.133	p = 0.887	p = 0.263	p = 0.533	p = 0.126	p = 0.5
Plate05A	10	p = 0.861	p = 0.821	p = 0.314	p = 0.761	p = 0.72	p = 0.5
Plate05B	10	p = 0.446	p = 0.129	p = 0.841	p = 0.614	p = 0.126	p = 0.5
Plate06A	10	p = 0.446	p = 0.345	p = 0.945	p = 0.939	p = 0.126	p = 0.5
Plate08	10	p = 0.266	p = 0.292	p = 0.11	p = 0.821	p = 0.126	p = 0.5
Plate08	15	p = 0.861	p = 0.982	p = 1	p = 0.533	p = 0.72	p = 0.5
Plate05A, Plate06A	10	p = 0.8	p = 0.201	p = 0.711	p = 0.692	p = 0.72	p = 0.5
Plate05A, Plate06A	20	p = 0.266	p = 0.684	p = 0.426	p = 0.91	p = 0.72	p = 0.5

*For large Poisson parameter, relatively fewer matrix entries are aggregated to reduce variation in the underlying parameter.
