# Supplementary material for: The Poisson distribution model fits UMI-based single-cell RNA-sequencing data
Source: BMC Bioinformatics. 2023 Jun 17;24:256. doi: 10.1186/s12859-023-05349-2 (PMC10276395; doi:10.1186/s12859-023-05349-2)
Supplement: Supplementary file 2 — Additional file 2. Supplementary figures including heatmaps and UMAP visualizations. [file 12859_2023_5349_MOESM2_ESM.pdf]

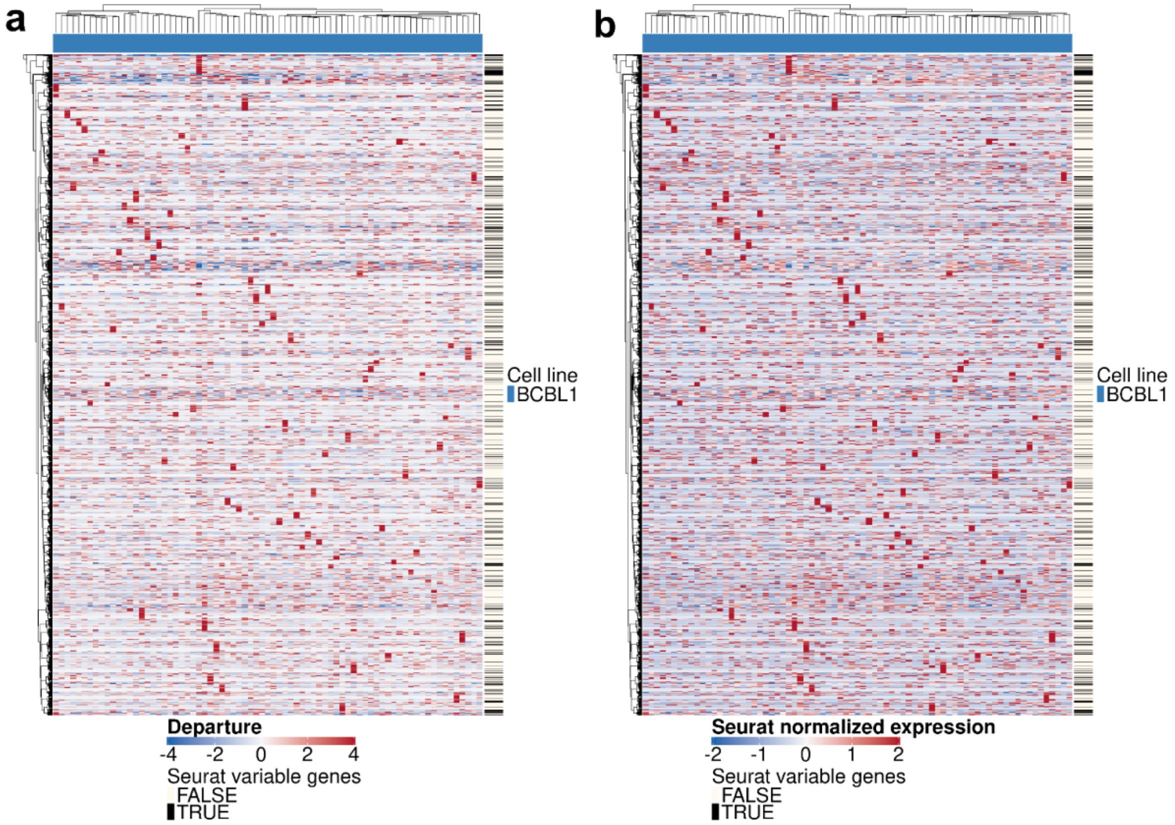

Figure S1: A heatmap view of the data representations of a single cell line data set (Plate 3). Data representations are based on (a) DIPD and (b) Seurat normalized and scaled counts before feature selection. The black colored lines in the sidebars on the right represent the top 2,000 most variable genes kept by the Seurat pipeline. Visually, both data representations demonstrate this data set is homogeneous.

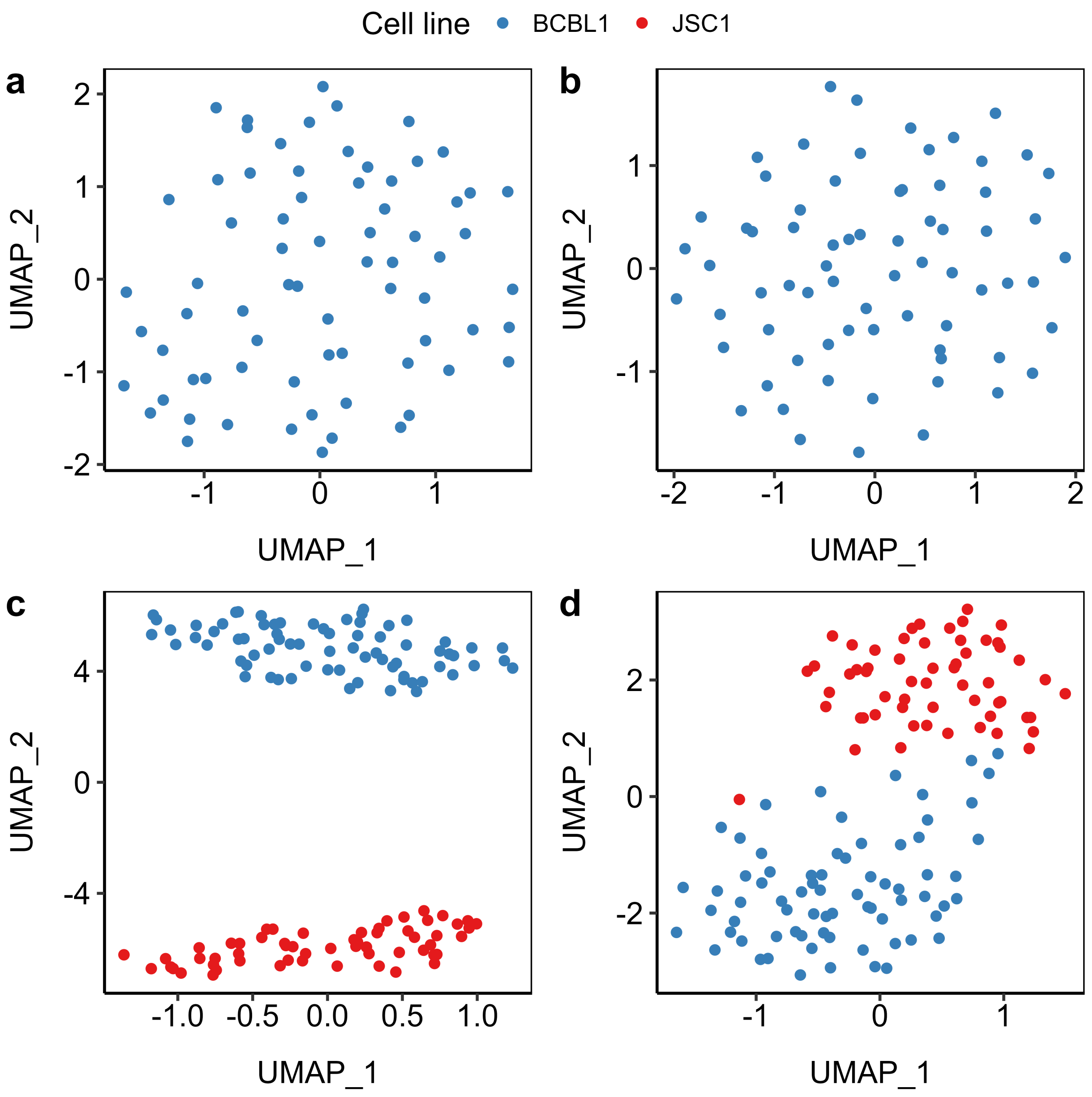

Figure S2: A UMAP view of the data representations of the clonal cell line data sets. Panels a and b depict a single cell line data set, while panels c and d show data set with two different cell lines. The data representations were generated using DIPD (panels a and c) and Seurat normalized and scaled counts before feature selection (panels b and d). The visualizations of panels a and b indicate that the data set is homogeneous, while the DIPD representation in panel c more effectively distinguishes between the two cell lines than the Seurat representation in panel d.

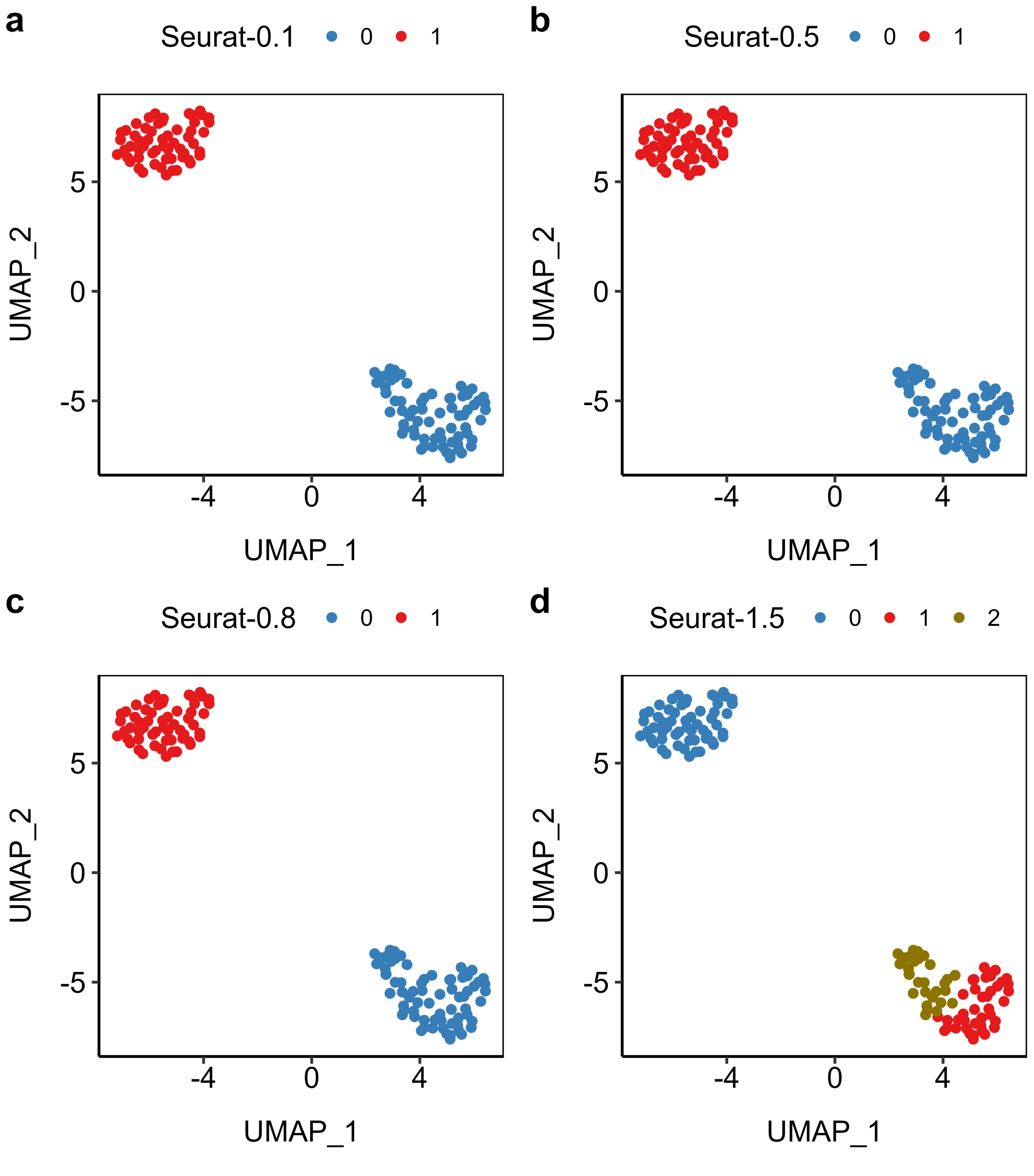

Figure S3: The Seurat clustering tuning process of the clonal cell line data sets in UMAP space. The input data (the same data set with two different cell lines as depicted in Figure S2) was generated using Seurat normalized and scaled counts, following feature selection and dimension reduction. The Seurat clustering pipeline produced accurate clustering outcomes within the resolution parameter range of 0.1 to 0.8. However, when the resolution parameter was set to 1.5, it resulted in an overestimation of the number of clusters.

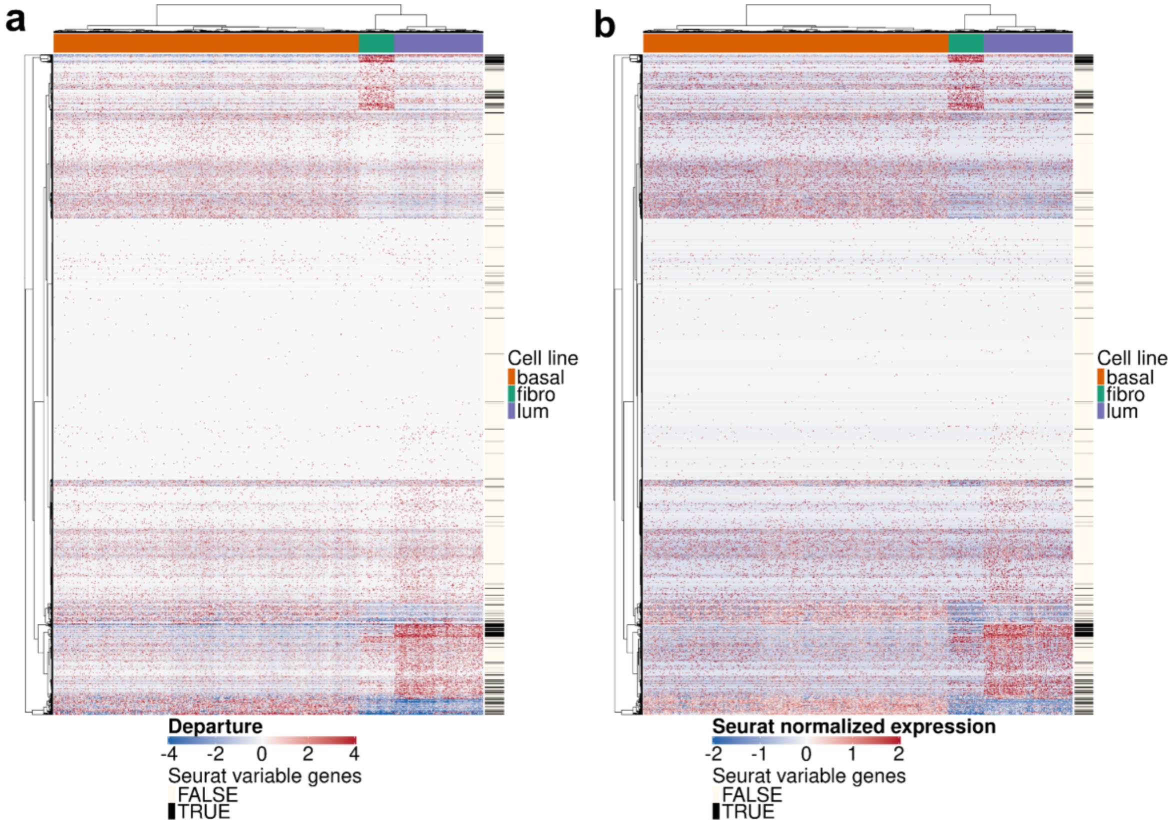

Figure S4: A heatmap view of the data representations of the three cell lines mixture data set. Data representations are based on (a) DIPD and (b) Seurat normalized and scaled counts before feature selection. The black colored lines in the sidebars on the right represent the top 2,000 most variable genes kept by the Seurat pipeline. Visually, both data representations effectively demonstrate the differentially expressed genes among the three cell lines. However, highly expressed genes within single cells, as depicted by the bright red spots, may potentially play a role in clustering but many are filtered out by Seurat.

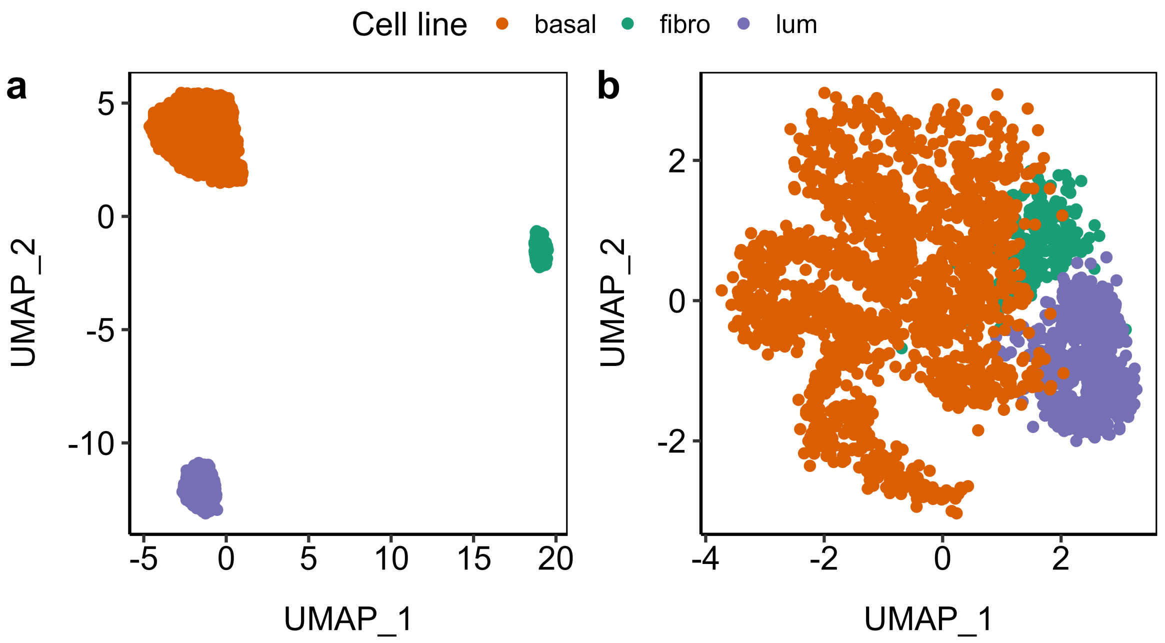

Figure S5: The UMAP visualization of the three cell lines mixture data set. The data representations are based on (a) DIPD and (b) Seurat normalized and scaled counts before feature selection. Visually, the DIPD representation more effectively distinguishes the three cell lines compared to the Seurat representation.

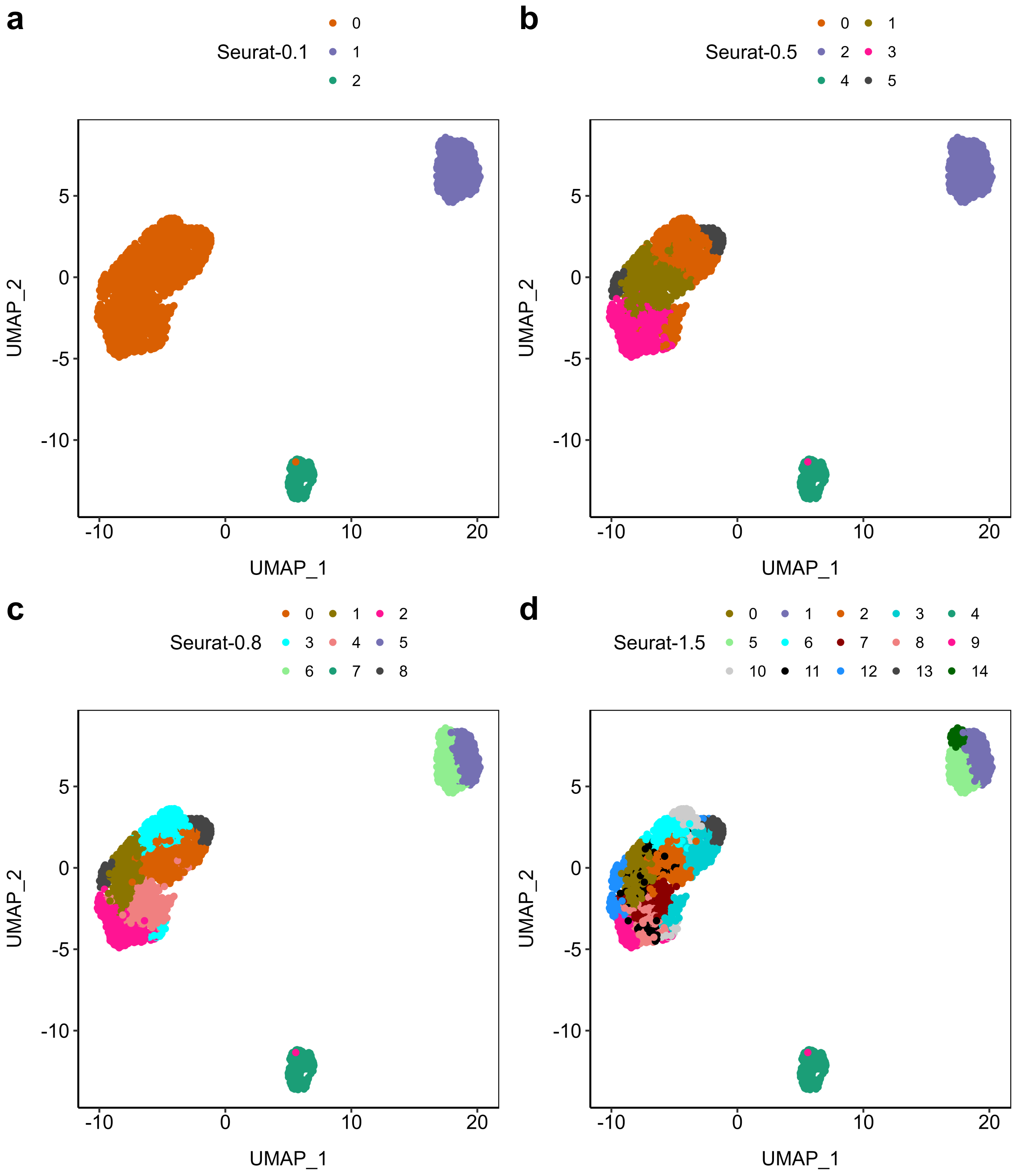

Figure S6: The Seurat clustering tuning process of the three cell lines mixture data set in UMAP space. The input data are generated using Seurat normalized and scaled counts, following feature selection and dimension reduction. The Seurat clustering pipeline produced accurate clustering outcomes only when the resolution parameter was set to 0.1. For other resolution parameter settings, it resulted in an overestimation of the number of clusters.

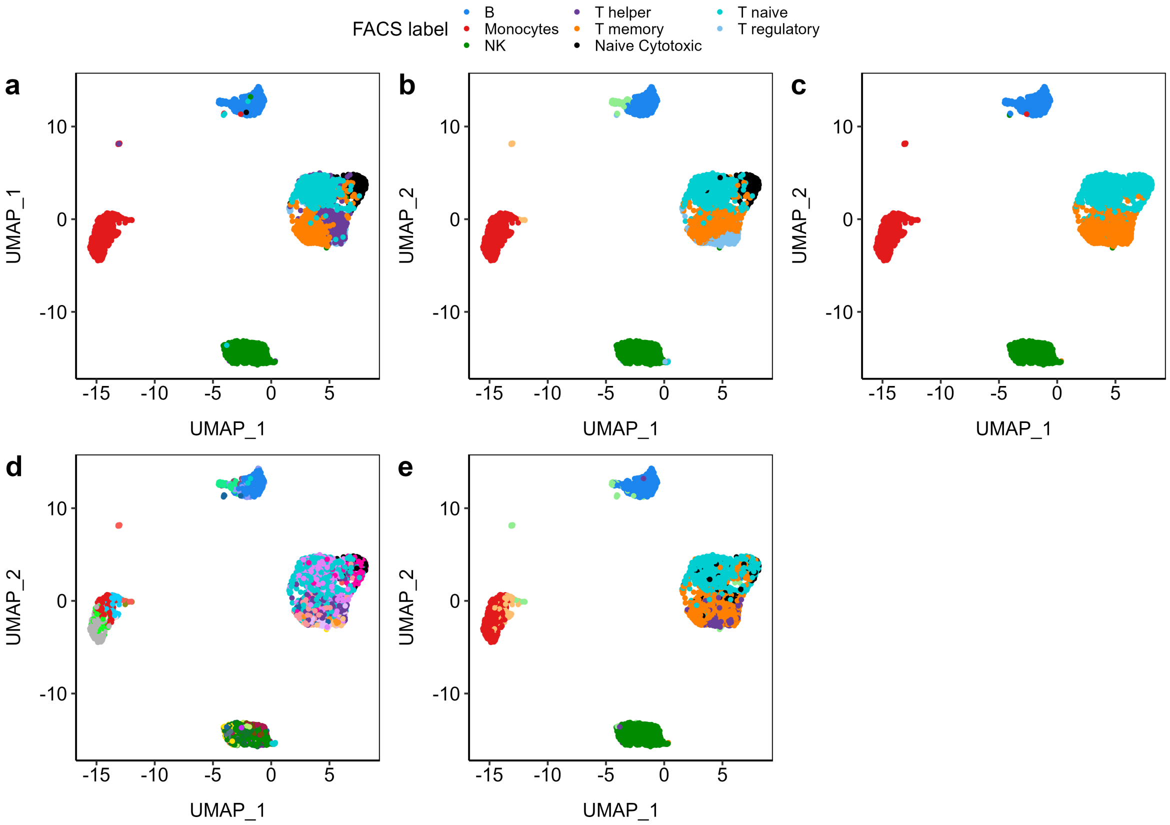

Figure S7: The UMAP visualizations of the clustering performance in the Zhengmix8eq data set. Panel a displays the FACS labels used as a benchmark to measure clustering performance. Panels b-e were generated using Seurat SCTransform, Monocle3, SC3, and TSCAN, respectively, with each color representing an identified cluster. Similar to the clustering results obtained from Seurat with log-normalized counts, these methods performed well in identifying the more distinct cell types (NK cells in green, monocytes in red, and B cells in blue), but failed to distinguish T cell subtypes.

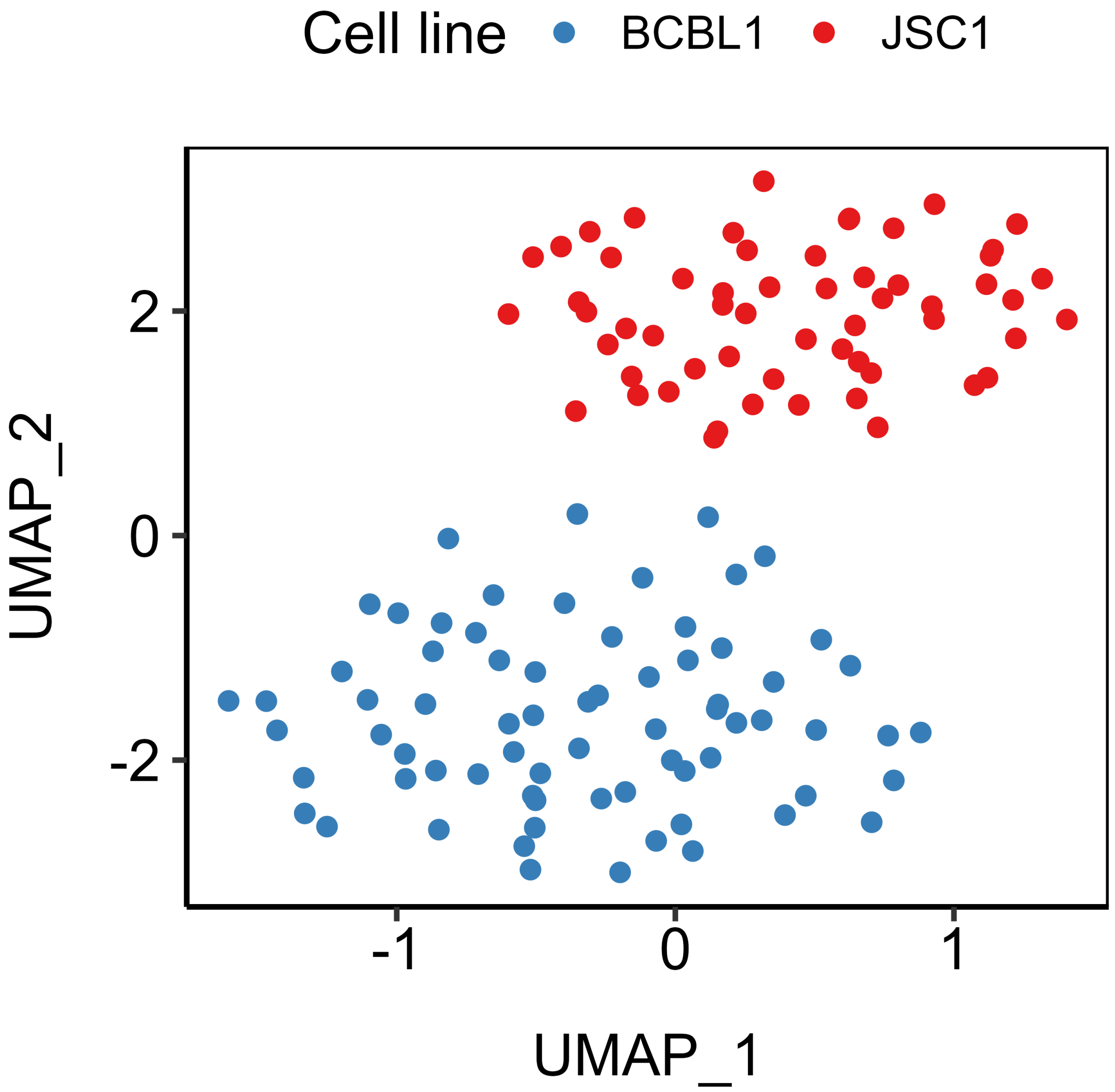

Figure S8: Clustering results of the clonal cell line data for two distinct cell lines in UMAP space. The data representation was generated using raw counts without UMI correction by DIPD. Our *Hclust-Departure* pipeline produced accurate clusters as anticipated. However, distinguishing between the two cell lines is not as evident as it is in the results obtained with UMI counts, as depicted in Figure S2, panel c.
