# Supplementary material for: The Poisson distribution model fits UMI-based single-cell RNA-sequencing data
Source: BMC Bioinformatics. 2023 Jun 17;24:256. doi: 10.1186/s12859-023-05349-2 (PMC10276395; doi:10.1186/s12859-023-05349-2)
Supplement: Supplementary file 3 — Additional file 3. The Q-Q envelope plot for small discrete counts. [file 12859_2023_5349_MOESM3_ESM.pdf]

Q-Q plot for small discrete counts

Visualization methods are useful for assessing the "Poissoneity" of scRNA-seq data. In general, the Q-Q (Quantile to Quantile) plot provides a useful visualization for comparing two distributions. These distributions can be either continuous or discrete, and a common application is to compare a data set represented by its Cumulative Distribution Function (CDF), with a hypothesized probability distribution, also represented by its (theoretical) CDF. The Q-Q plot shows the respective quantiles (the input or argument of the two CDFs) on the vertical and horizontal axes, corresponding to all the probabilities between 0 and 1. The closeness of the graph to the 45° line indicates the closeness of the two probability distributions.

For discrete distributions that take on only small integer values, such as those commonly encountered in scRNA-seq data, conventional Q-Q plots may not be effective. This is illustrated in Additional file 3, using an example of two very discrete distributions, P and Q. To define P and Q, we use the notation p_i and q_i to denote the probability of getting i in distribution P and Q, respectively. P is defined as $p_0 = 1/3$, $p_1 = 1/2$, $p_2 = 1/6$ (shown as black in Figure S9 panel a), and Q is defined as $q_0 = 2/3$ and $q_1 = 1/3$ (shown as black in Figure S9 panel d). In panels b and e, we can observe the corresponding CDFs for P and Q, respectively. These CDFs are shown as step functions, with steps at the half integers, where the height of each rectangle represents the corresponding probability. Due to the strongly discrete nature of these distributions, the standard Q-Q plot (shown as black dots in panel h in Figure S9) for the distribution P on the vertical axis, and distribution Q on the horizontal axis, is challenging to interpret visually. They do reflect the few integer values taken on by these random variables, but essentially ignore the important probabilities driving the difference between these distributions. Note that in practice, p_i and q_i can either be values from a theoretical distribution such as the Poisson, or can represent empirical probabilities derived from count data as proportions.

We provided a more informative version of the Q-Q plot by using the idea of *continuity correction*, which provides a useful bridge between continuous and discrete distributions. This idea has been fundamental to the Normal approximation of the Binomial. The main idea was to approximate an integer-valued discrete distribution, with a continuous probability distribution. In this manner, the point mass at each integer i is replaced by a bar on the interval $[i - \frac{1}{2}, i + \frac{1}{2}]$ with height p_i for distribution P (as depicted by the blue shaded area in panel a) or q_i for distribution Q (as depicted by the green shaded area in panel d). The CDF of a continuity-corrected discrete distribution was shown to be a piecewise linear function with knots at the half integers, essentially a linear interpolation. This is illustrated by the shaded areas in panels b and e. The corresponding quantiles of the distributions are shown in panels c and f.

To ensure a proper comparison of the probability distributions of two samples, the straightforward pairing of sorted values is not applicable as they have varying CDF values (shown in the horizontal axis in panels c and f). Therefore, an interpolation process is required to ensure that both samples share the same common CDF values. This involves applying linear interpolation to each unique CDF value that has an original data point in one sample but lacks a corresponding point in the other sample. The resulting common CDF values after interpolation should possess unique points that align with the unique CDF values from either sample. The union of the knots of the CDFs for distributions P and Q is displayed in panel g, while panel h of Figure S9 exhibits the Q-Q plot comparing respective quantiles of the two distributions (P and Q) as the orange curve. As both CDFs are piecewise linear, this curve is also piecewise linear with knots at the union of the CDF knots.

In the context of comparing an empirical CDF with a theoretical model CDF, the *Q-Q envelope* is a helpful tool for understanding the natural variability in a Q-Q plot. This approach models the sampling process by simulating multiple samples of the same size from the candidate theoretical distribution and overlaying the envelope of resulting CDFs (using the continuity correction technique). By doing so, the *Q-Q envelope* can differentiate between observed patterns that are significant and those that are simply artifacts of sampling variation. In low-count discrete settings, conventional Q-Q plots may not be useful, but as demonstrated in the Results section, continuity-corrected Q-Q envelopes are very informative.

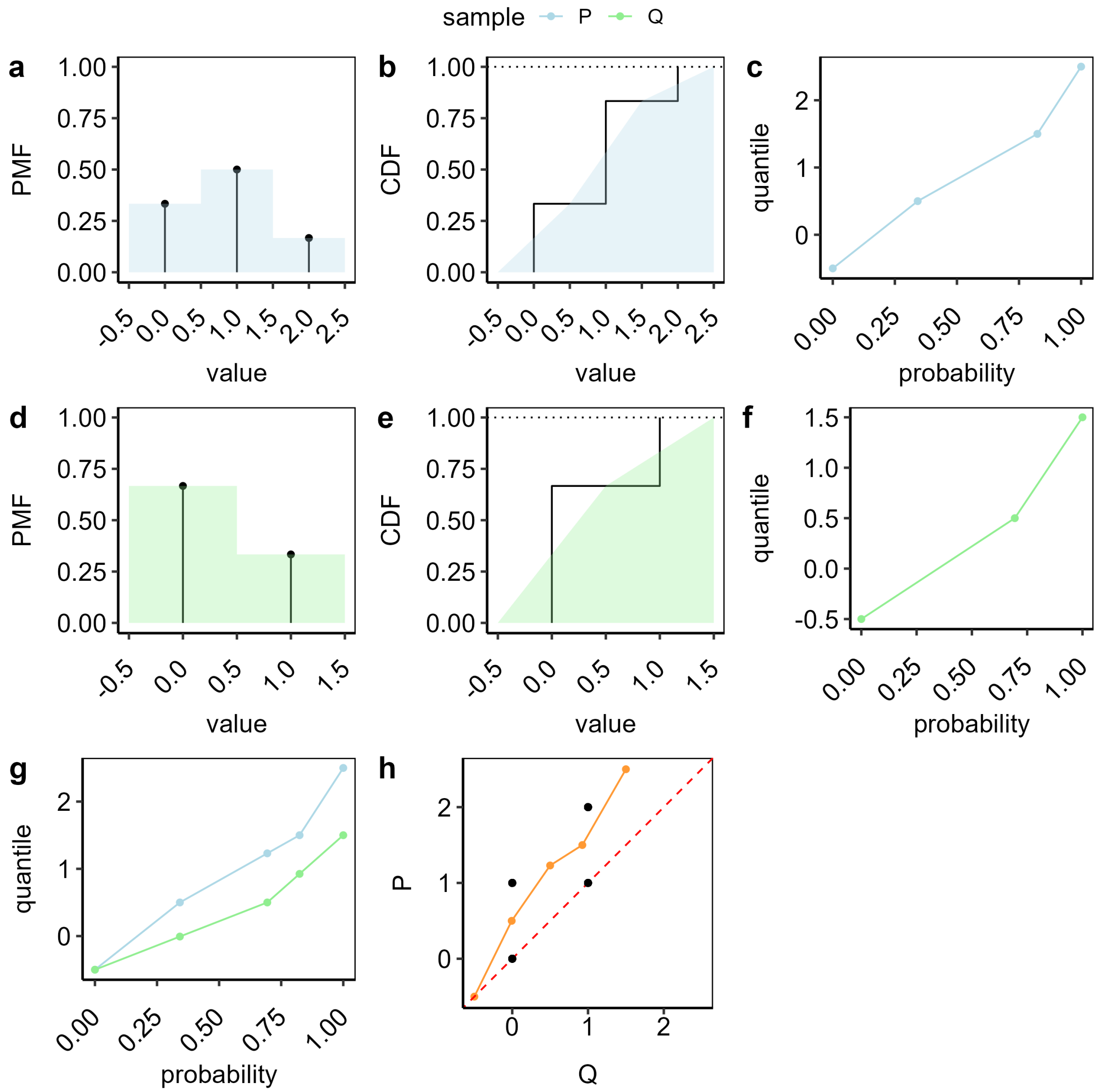

Figure S9: *Continuity correction* for point mass function (PMF) and Q-Q plot developed for small discrete counts. (a) Point mass function (PMF) for distribution P. (b) Cumulative distribution function (CDF) for distribution P, with the blue shaded area representing the continuous approximation. (c) Corresponding quantiles from distribution P. (d) PMF for distribution Q. (e) CDF for distribution Q, with the green shaded area representing the continuous approximation. (f) Corresponding quantiles from distribution Q. (g) Corresponding quantiles from distributions P (blue) and Q (green) after *Continuity correction* and linear interpolation. (h) Q-Q plot comparing two discrete distributions P and Q. The black dots show all the conventional Q-Q points, typically piled up at a few small integers. The orange curve is the corresponding Q-Q plot after applying *continuity correction* and linear interpolation, providing a more informative way of comparing distributions with small discrete counts.
