# Supplementary material for: The Poisson distribution model fits UMI-based single-cell RNA-sequencing data
Source: BMC Bioinformatics. 2023 Jun 17;24:256. doi: 10.1186/s12859-023-05349-2 (PMC10276395; doi:10.1186/s12859-023-05349-2)
Supplement: Supplementary file 4 — Additional file 4. The summary of the computational time required for implementing DIPD on the datasets used in this manuscript. [file 12859_2023_5349_MOESM4_ESM.pdf]

Table S4: Summary of computational time for DIPD

Data set	Size	Computation time
Single clonal cell line data (Plate03)	71 cells, 11,227 genes	3.2s
Single clonal cell line data (Plate05A, Plate06A)	129 cells, 12,167 genes	6.7s
Three cell lines mixture data	2,609 cells, 21,247 genes	250.6s
Multiple cell lineages data	1476 cells, 12,822 genes	71.2s
PBMC data (Zhengmix8eq)	3994 cells, 15,716 genes	284.7s

*The DIPD algorithm was implemented in R Studio version 4.2.1 on a server with 64GB of RAM and an Intel(R) Core(TM) i9 processor.
